# The Minimal Translation Machinery: What We Can Learn From Naturally and Experimentally Reduced Genomes

**DOI:** 10.3389/fmicb.2022.858983

**Published:** 2022-04-11

**Authors:** María José Garzón, Mariana Reyes-Prieto, Rosario Gil

**Affiliations:** ^1^Departament de Genètica, Universitat de València, Burjassot, Spain; ^2^Institute for Integrative Systems Biology, Universitat de València–Consejo Superior de Investigaciones Científicas, Paterna, Spain; ^3^Sequencing and Bioinformatics Service, Foundation for the Promotion of Sanitary and Biomedical Research of the Valencian Community, Valencia, Spain

**Keywords:** translation machinery, minimal genome, endosymbionts, JCVI-sync3.0, cosymbionts

## Abstract

The current theoretical proposals of minimal genomes have not attempted to outline the essential machinery for proper translation in cells. Here, we present a proposal of a minimal translation machinery based on (1) a comparative analysis of bacterial genomes of insects’ endosymbionts using a machine learning classification algorithm, (2) the empiric genomic information obtained from *Mycoplasma mycoides* JCVI-syn3.0 the first minimal bacterial genome obtained by design and synthesis, and (3) a detailed functional analysis of the candidate genes based on essentiality according to the DEG database (*Escherichia coli* and *Bacillus subtilis*) and the literature. This proposed minimal translational machinery is composed by 142 genes which must be present in any synthetic prokaryotic cell designed for biotechnological purposes, 76.8% of which are shared with JCVI-syn3.0. Eight additional genes were manually included in the proposal for a proper and efficient translation.

## Introduction

The minimal genome was originally defined as the set of genes necessary and sufficient for life under low restrictive conditions (Mushegian, 1999). Therefore, a minimal genome must guarantee the three functional pillars of a living cell (Gil, 2014). First, a simplified DNA replication and repair system, as well as transcription and translation systems, to ensure the maintenance and the proper use of its genetic information; second, a self-sufficient metabolism that meets basic energy and structural requirements; last, an envelope that shelters all the cellular machinery and allows interaction with the environment, as well as the generation of descendants.

Many authors have striven to define the minimal genome to understand the basic principles of life and to apply it for biotechnological purposes (Gil, 2014; Hutchison et al., 2016; Ziegler and Takors, 2019). One of the most used strategies to define the minimal genome is based on the study of organisms with naturally reduced genomes due to their living style in association with a eukaryotic host, no matter if the association is mutualistic or parasitic (Moya et al., 2008). On account of this host-dependence characteristic and the niches in which they thrive, it has always been a challenge to apply experimental techniques to investigate their physiological processes in real-time. Nevertheless, thanks to the development of high-throughput sequencing technologies, many genomes of mutualistic insect endosymbionts have been sequenced and are available for in depth studies. These bacteria have been recognized as a key factor in the evolutionary success of this group of animals (Moya et al., 2008), as they provide their hosts with tools to adapt to new environments, in many cases related to nutrient provision. During the process of symbiotic integration, the endosymbiont genomes undergo what has been called the “genome-reduction syndrome” (Gil et al., 2010). The genome size of obligate (primary) endosymbionts (OS) can vary, depending on the age of the association and the degree of symbiotic integration achieved, leading to small genomes (circa 600 kb in many cases) or even to tiny genomes (Moran and Bennett, 2014), also known as “symbionelles” (Reyes-Prieto et al., 2014), as it is the case of the 106-kb genome of “*Candidatus* Hodgkinia cicadicola” str. TETCHI4, the smallest sequenced bacterial genome to date (Łukasik et al., 2018). The strong genome shrinkage undergone by these tiny genomes, has gone beyond the limit of what has been defined as a minimal genome (Gil, 2014), which contemplates about 187–205 protein-coding and 35–38 RNA genes. Although this proposal includes universally retained genes, and some persistent ones [i.e., non-ubiquitous genes conserved in most genomes, therefore, non-essential but needed for robust long-term survival; Acevedo-Rocha et al. (2013)], it is still a theoretical proposal that has never been proven to be enough to maintain a living cell.

**FIGURE 1:**
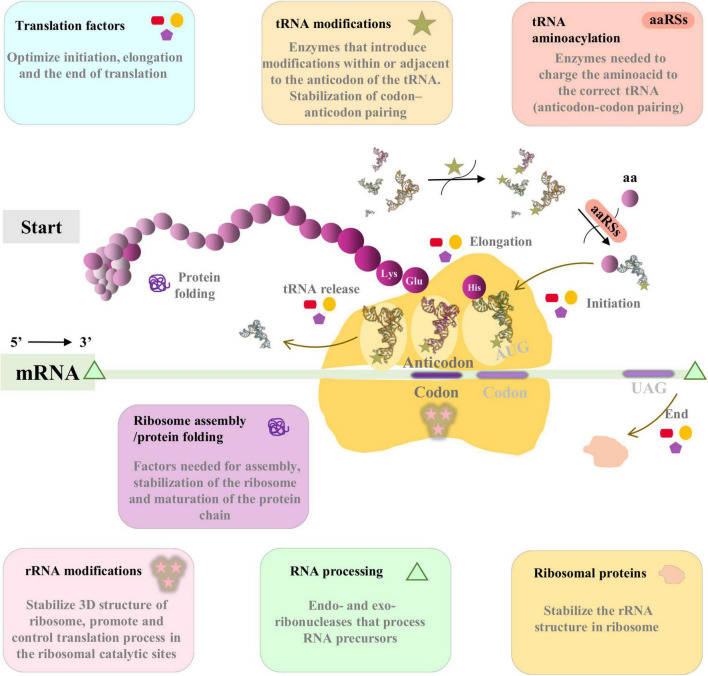
Simplified schematic view of translation in bacteria. In this work all the proteins included in translation machinery are classified in seven functional categories: ribosomal proteins (orange), tRNA aminoacylation (coral), rRNA modifications (pink), tRNA modifications (wheat), ribosome assembly/protein folding (orchid), Translation factors (blue), and RNA processing (green). The most important functions are summarized in the respective panels of each category. The tRNA structure corresponds to tRNA(Ile) from PDB database (1FFY; Silvian et al., 1999), and has been modified with PyMOL software (Schrödinger and DeLano, 2020) for this figure.

The translational machinery is essential for the maintenance and continuity of the cell and is, by far, the most complex part of modern cells (Figure 1). It is made of numerous macromolecules, including proteins and RNAs (mRNAs, tRNAs, rRNAs, and other small RNAs), all of which are encoded in an organism’s genome. Most genes involved in translation are considered essential or quasi-essential for cell survival. Remarkably, there are many examples of endosymbiotic bacteria that lack important translational genes. In such cases, it has been hypothesized that it must be the host, or a co-obligate endosymbiont where appropriate, which provides the informational precursors to the endosymbiont which holds the deficiency (Sloan and Moran, 2012).

In 2010, the 1,079-kb genome of *M. mycoides* JCVI-syn1.0 was chemically synthesized and its cell growth when transplanted into the cytoplasm of *Mycoplasma capricolum* was proven (Gibson et al., 2010). The first semisynthetic organism based on modern living cells, was created. Afterwards, using JCVI-syn1.0 as a starting point, and by removing non-essential genomic regions through a cyclic design-build-test (DBT) strategy, Hutchison and co-workers managed to obtain *M. mycoides* JCVI-syn3.0 (Hutchison et al., 2016). This semisynthetic organism is viable in axenic culture and its streamlined genome contains 438 protein-coding and 35 RNA genes. It derives from a Mollicutes, which have evolved from ancestral Gram-positive bacteria (Parks et al., 2018) for which the translation machinery has been extensively studied in the last decade (Grosjean et al., 2014). In their work, Grosjean and collaborators identified translation-related protein-coding genes shared by 39 selected Mollicutes’ genomes and compared them with those of *Escherichia coli* and *Bacillus subtilis*, as Gram-negative and Gram-positive bacterial models, respectively. A set of 260 protein-coding genes involved in translational functions were selected for the study and classified in the following functional categories: ribosomal proteins, tRNA aminoacylation, rRNA modifications, tRNA modifications, ribosome assembly, translation, and RNA processing. They found that the categories of aminoacyl-tRNA synthetases, ribosomal proteins and translation factors contained the most preserved genes, while some enzymes involved in specific modifications of tRNAs, 16S rRNA, and 23S rRNA, fundamental for decoding and peptidyl transfer, were also essential.

The updated proposal of the minimal genome by Gil (2014) included a revised version of the genes involved in translation. It is worth mentioning that, among the genes included in the previously defined core of the minimal genome (Gil et al., 2004), four of the poorly characterized genes due to the lack of information at that time, were later identified as part of the translation machinery (i.e., *rsm*H, *rsm*I, *til*S, and *ybe*Y). Twelve additional persistent genes were added to the new version, many of which encode ribosomal proteins that, due to their small size, might have been missed during genome annotation or lost in extremely reduced genomes (Nikolaeva et al., 2021).

For this work, in an attempt to get closer to the universal core of the minimal translation machinery, we selected most of the complete reduced genomes of insect endosymbionts available in the SymGenDB database by 2020 (Reyes-Prieto et al., 2020), to search through an unsupervised machine learning technique (hierarchical clustering) for those essential and persistent genes involved in translation. Then, in order to validate our *in silico* minimal translation machinery proposal, we compared it with that of *M. mycoides* JCVI-syn3.0. Finally, we compared the obtained translation machinery with the one defined by Grosjean et al. (2014) for Mollicutes. Our final proposal is composed of 142 protein-coding genes and defines the protein components of the minimal translation machinery that must be present in a hypothetical viable prokaryotic cell, which can be useful for defining a biological chassis to which desired functions can be added for biotechnological purposes.

## Materials and Methods

### Bacterial Genomes Used in This Study

The endosymbiont genomes to be included in the analyses and their accession IDs were mostly retrieved from SymGenDB (Reyes-Prieto et al., 2020). We added the following genomes that were not available in SymGenDB (2020) due to their posterior discovery or annotation, or because they are not listed in the KEGG database: “*Candidatus* Serratia symbiotica” SeCistrobi, “*Candidatus* Tremblaya phenacola” PPER, “*Candidatus* Tremblaya princeps” TPPLON, *Cardinium* cSfur, *Cardinium hertigii* cBtQ1, *Neisseria meningitidis* MC58, *Serratia symbiotica* SCt-VLC, “*Candidatus* Tremblaya princeps” TPMHIR1, “*Candidatus* Tremblaya princeps” TPPMAR1, “*Candidatus* Tremblaya princeps” TPFVIR, “*Candidatus* Tremblaya princeps” TPTPER1, “*Candidatus* Sulcia muelleri” TETUND, “*Candidatus* Hodgkinia cicadicola” TETUND2, *Buchnera aphidicola* BCc and “*Candidatus* Sodalis sp.” SoCistrobi. Their genomic data were retrieved automatically on August 2020 using the efetch command from GeneBank,^1^ except for “*Candidatus* Tremblaya phenacola” PPER, *Serratia symbiotica* SCt-VLC and *Cardinium hertigii* cBtQ1, which were manually downloaded because only shotgun assemblies are available. Additionally, in order to generate a complete universe of translation-involved genes, we included in our analysis 10 bacterial genomes with no reduction, two of them, *E. coli* and *B. subtilis*, are common bacterial models for Gram-negative and Gram-positive bacteria, respectively. The other eight were selected because they are taxonomically diverse and can be grown in the laboratory in axenic culture. The Prokka software tool (Seemann, 2014) was used to re-annotate all genomes for homogeneous results. Finally, we also included in our comparisons the genome of *M. mycoides* JCVI-syn3.0. This genome annotation was retrieved from Hutchison et al. (2016). We only took into consideration genes classified as ribosome biogenesis, RNA metabolism, protein folding, translation, RNA, rRNA modification, tRNA modification, and regulation. The sequences of ORFs classified as “unclear category” were used to perform a BLASTP against the non-redundant protein sequences database at the NCBI web (The Blast Sequence Analysis Tool, 2022) to look for putative functions of the hypothetical conserved proteins they might encode. All 110 bacterial genomes under study have been compiled in a dataset called “cosym” with 92 entries as a result of considering coprimary insect endosymbionts (symbiotic consortia) as single entities (Supplementary Material 1), and a list of the genera included in this study is listed in Table 1.

**TABLE 1:** Genera of the symbionts and free-living bacteria whose genomes have been used in this study.

Symbionts	
*Arsenophonus* (1)	*Riesia* (2)
*Baumannia* (2)	*Serratia* (3)
*Blattabacterium* (8)	*Sodalis* (3)
*Blochmannia* (6)	*Sulcia* (8)
*Buchnera* (19)	*Tachikawaea* (1)
*Carsonella* (7)	*Tremblaya* (8)
*Doolittlea* (1)	*Uzinura* (1)
*Evansia* (1)	*Walczuchella* (1)
*Gullanella* (1)	*Zinderia* (1)
*Hoaglandella* (1)	*Cardinium* (3)
*Hodgkinia* (3)	*Legionella* (1)
*Mikella* (1)	*Neisseria* (1)
*Moranella* (1)	*Rickettsia* (1)
*Nasuia* (1)	*Wigglesworthia* (2)
*Pantoea* (1)	*Wolbachia* (6)
*Profftella* (1)	Non-genera, Secondary endosymbiont of (3)
**Free-living**	
*Achromobacter* (1)	*Mesorhizobium* (1)
*Bacillus* (1)	*Pseudomonas* (1)
*Caulobacter* (1)	*Serratia* (1)
*Escherichia* (1)	*Sphingobacterium* (1)
*Flavobacterium* (2)	

*In brackets, the number of genomes within the same genera.*

### Identification of the Translational Gene Sets and Gene Orthology Analysis

Genes were classified into functional categories based on the previous work by Grosjean et al. (2014). We defined the set of translational protein-coding genes (*translational gene set*) of the two model species considered through several steps. First, we retrieved the genes from *E. coli* and *B. subtilis* that have been included in the work by Grosjean et al. (2014) as encoding essential components of the translation machinery. Then, we searched for selected GeneOntology (GO) terms on UniProt (UniProt Consortium, 2015) and EcoCyc (Keseler et al., 2013), in order to update the list (Supplementary Material 2). The GO terms included were: 0000154 (rRNA modification), 0001510 (RNA methylation), 0001680 (Addition of CCA 3’-end of tRNA), 0005840 (Ribosome), 0006364 (rRNA processing), 0006396 (RNA processing), 0006400 (tRNA modification), 0006412 (Translation), 0006417 (Translational regulation), 0006457 (Protein folding), 0008033 (tRNA processing), 0009451 (RNA modification), 0042255 (Ribosomal assembly), and 0042254 (Ribosomal biogenesis). In this step, a manual curation of gene names was mandatory to eliminate duplicated candidates (i.e., the same ortholog with different annotated names), to use the UniProt accepted nomenclature for genes with a double translational function, and to remove genes not strictly related to translation.

Orthologous genes (paralogs included) in all genomes under study were identified using the Roary software (Page et al., 2015) with default parameters. The absence (0 count) or presence (1 count) of each one of these orthologs in each genome was counted, creating several matrices for further analyses, one per each functional translation category (Supplementary Material 3).

### Classification of Genes and Statistical Analysis

Hierarchical cluster analysis (HCA), an unsupervised machine learning approach for grouping datasets into clusters, was used for the classification of the dataset genes. HCA was performed in R with gplots:heatmap.2 and stats:hclust (complete method) functions. The input data were the presence/absence matrices obtained in the gene orthology analysis. To extract the gene names of the dendrogram clustering, the cutree function of the stats R-package was used. The treatment of the data, the construction of figures and the statistical analyses performed for this work were carried out with *ad doc* scripting in RStudio 4.0.3 using the stats, ggplot2 (Wickham, 2016) and ggthemes R packages. The full script is available through https://github.com/majogarzon/MinTransMach.git.

## Results

### Selection of the Bacterial Genomes for the Study and Characterization of Their Pangenome

Defining the basic living functions to generate a simplified bacterium that might be modulated under laboratory conditions with desirable and predictable outcomes for biotechnological purposes is a great challenge. The naturally reduced genomes of insect endosymbionts have historically been studied to approach the minimal genome concept, providing valuable information about those functional modules that are necessary and sufficient for life. Previous comparative studies (Gil et al., 2004) concluded that the minimal genome is substantially enriched in genes involved in genetic-information processing, mainly coding for the elements of the translational apparatus, the most complex machinery in a living cell. Yet, it has been possible to define simplified but still functional translation machineries after a reductive evolution in Mollicutes and insect endosymbionts (Grosjean et al., 2014; Gil and Peretó, 2015).

In this work, to further explore and validate the minimal translation machinery, our search began with the selection of organisms with naturally reduced genomes to compare them with known free-living bacterial models which must possess efficient, complete, and more complex translational apparatus. As a starting point, taking advantage of the availability of extensive genomic information and the bioinformatic tools developed in recent years, we retrieved all the genomes annotated as insect endosymbionts from SymGenDB, a database that lodges genomic information of organisms involved in symbiotic relationships (Reyes-Prieto et al., 2020), and updated the information by manually including some additional endosymbiont genomes, as described in Materials and Methods. All these genomes were classified depending on their symbiotic relationship as primary or obligate symbionts (OS) when they are necessary for the survival and reproduction of the host, and as secondary symbionts (SS) when they maintain a facultative symbiotic relationship in terms of survival. Furthermore, we included several non-symbiotic organisms, designated as free-living (FL), to have a complete representation of the universe of translational genes in our data set. All the 110 organisms used in our analyses are listed along with taxonomic and genomic information in Supplementary Material 1, and a summary of their genera is listed in Table 1.

Many insects live in obligate association with more than one endosymbiotic bacterium. It has been observed that the presence of two (or more) co-primary endosymbionts allows a greater reduction of the bacterial genomes, far below the definition of a minimal genome (Sloan and Moran, 2012). Probably this means that they can complement each other by exchanging some gene products to perform essential functions (Reyes-Prieto et al., 2014), including informational ones (i.e., DNA replication, transcription and translation). For this reason, to generate our final genome dataset we considered all coprimary symbionts as a single entity (consortia sheet in Supplementary Material 1), leading to only 92 entries. Next, we searched for the orthologs in the genome’s dataset using the Roary software. We found 101,012 clusters of orthologs which compose the whole pangenome of the organisms under study.

### Computational Search of the Universe of Genes for Translation

Before determining our universe of translational genes, we had to cope with the existence of poorly or wrongly annotated genes and pseudogenes within the analyzed genomes. In fact, a critical difficulty in carrying out this work has been the automation of the process because, even though great efforts are being made to unify the nomenclature (as defined by the International Nucleotide Sequence Database Collaboration, INSDC; Brunak et al., 2002), at present no database provides the unified names for all genes. Most gene descriptions are still based on their initial identification by classical genetics in each given organism, and a recent discovery of their function is often associated to errors, such as entering a function twice without unified descriptors or simply by not including it in databases. For this work, the UniProt nomenclature recommended by the INSDC has been used; in cases where there was no classification, the accepted nomenclature for in *E. coli* K-12 MG1655 (taxonomy ID 511145) was chosen (Schoch et al., 2020).

**FIGURE 2:**
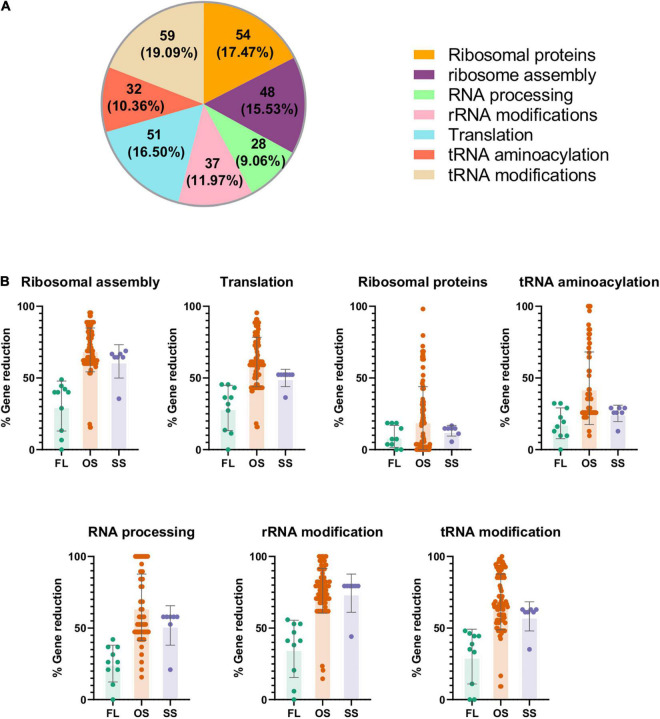
Representation and analysis of the universe of translational genes in the data under study. The complete list of genes can be found in Supplementary Material 2. **(A)** Translational set composed by 309 genes after manual curation. **(B)** Gene reduction level of the genomes under study measured as percentage of translational orthologs not detected in each organism related to the maximum number of genes found in each translational category of the dataset. FL: free-living. OS: obligate endosymbiont. SS: secondary symbiont.

Once this problem was solved, we set up a *universe of translational protein-coding genes*, which is composed by the genes that encode the proteins that integrate the translation apparatus, as well as those that directly or indirectly participate in the different stages of rRNA or tRNA processing. This gene set was defined based on the genomes of the two selected model bacteria, *E. coli* K-12 MG1655 and *B. subtilis* 168. We detected a total of 309 unique genes (Supplementary Material 2). Most of them belong to the ribosomal proteins and tRNA modification categories, and only a few of them to RNA processing (Figure 2A).

Then, we compared the previously defined pangenome to the universe of translational genes, to search for the 309 genes involved in translation. As expected, the genome reduction process affects the total number of genes for this function, and the losses depend on the translational subprocess involved (Figure 2B). Most ribosomal proteins are present in all organisms regardless of their lifestyle, confirming the importance of the whole ribosome as a functional unit. In contrast, many genes of the other translational categories have been lost, especially in OS and, to a lesser extent, in SS, certainly affected by the genome reduction syndrome. The less conserved genes belong to the ribosome assembly and rRNA/tRNA modification categories, as previously described (de Crécy-Lagard, 2007; Grosjean et al., 2014).

Next, we explored the datasets for essential and persistent genes (Acevedo-Rocha et al., 2013) to get closer to the definition of a functional minimized translation machinery. Evidently, if all organisms have an ortholog of a given gene, its function must be essential and must be included in the minimal translation machinery. Conversely, a gene that only presents orthologs in few organisms with reduced genomes, would not be essential. Based on this notion, a hierarchical clustering analysis was carried out, grouping the data in each of the seven translational subprocesses considered. This machine-learning unsupervised classification algorithm managed to separate each subset of data into two clusters (Supplementary Figure 1). A total of 134 orthologs were found in the seven clusters with higher counts and represent the candidate genes to be included in a first proposal of a minimal translational machinery (Supplementary Material 4). In previous works, *Buchnera aphidicola BCc* the OS of the aphid *Cinara cedri* (Pérez-Brocal et al., 2006; abbreviated as *bcc* in our study) was considered to possess a small genome close to what could be considered a minimal genome, still able to support the translation process autonomously, while tiny genomes were those that had already lost this ability, so that even genes essential to the process had been lost, making them dependent on the cooperation of a cosymbiont, or even the host, to perform translation (Reyes-Prieto et al., 2014). Therefore, as a first proxy for validation of our approach to define a minimal translational machinery, we compared our results with the genes from the *bcc* genome as a naturally minimized reference genome. The *bcc* genome retains around half of the universe of translational orthologs (150 out of 309 genes), of which it shares 125 genes with our first minimal proposal. Additionally, our proposal contains nine genes (*queA*, *rlmB*, *rne*, *rnhA*, *rplR*, *tgt*, *trmB*, *tsaC*, and *tusE*) that are not present in *bcc*. As *bcc* is cosymbiont of *Serratia symbiotica* str. “Cinara cedri” (SS of *Cinara cedri*; abbreviated *ssz*), we searched for those genes in the cosymbiont’s genome. The presence of all nine genes in *ssz* indicates that it probably contributes essential translation genes to the symbiotic relationship. On the other side, the 25 additional genes present in *bcc* and absent in our minimal proposal might reflect that its genome reduction is still an ongoing process. However, as 14 out of these 25 genes are essential in *E. coli* (see next section) and belong to all possible translational subcategories, it cannot be ruled out that some of them are necessary to improve the efficiency of translation in the specific intracellular environment of this bacterium.

### Refining the Minimal Translation Machinery

**FIGURE 3:**
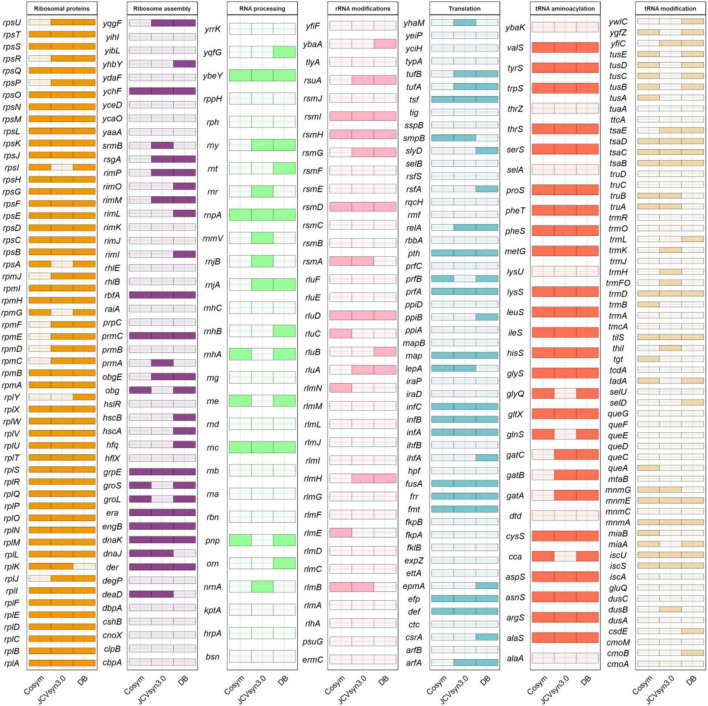
Comparison of translational genes in DataBases [bacterial gene list of essential translational genes for *Escherichia coli* K-12 MG1655 and *Bacillus subtilis* 168, obtained from the Database of Essential Genes, Subtiwiki, Goodall et al. (2020) and Koo et al. (2017)], cosym (dataset of candidate genes included in our preliminary proposal of a minimal translational machinery obtained from endosymbiont genomes by HCA in this work) and JCVI-syn3.0 (dataset of genes annotated in this genome as involved in translation).

In order to test the viability of our first proposal, we compared it with the set of essential genes involved in translation of *E. coli* K-12 MG1655 and *B. subtilis* 168, according to the DEG database (Luo et al., 2021) and more recent studies on essentiality in *B. subtilis* (Koo et al., 2017; Pedreira et al., 2022) and *E. coli* (Goodall et al., 2020), and with the synthetic JCVI-syn3.0 organism (Hutchison et al., 2016). These two comparisons provide complementary information. While the DEG database reports those genes indispensable for the immediate survival of an organism, the information obtained from JCVI-syn3.0 also highlights the importance of persistent genes, needed for the cell to maintain itself for an extended term. Hutchison et al. (2016) indicate that the JCVI-syn3.0 genome has 195 genes for genetic information storage and processing. Among them, we found that 144 genes are involved in translational processes. Additionally, because 92 genes without assigned biological function were predicted when the JCVI-syn3.0 genome was published (Hutchison et al., 2016), we searched for putative functions of this last set of genes by BLASTP against the non-redundant protein sequences (nr) database from the NCBI web page. Several possibly interesting enzymes were found: a bifunctional oligoribonuclease and PAP phosphatase (*nrnA;* EC 3.1.3.7), a putative pre-16S rRNA nuclease RNaseH-like (*yqgF;* EC 3.1.-.-) and a ribosomal L7Ae/L30e/S12e/Gadd45 family protein (EC 3.1.26.5). The later must be a *rpmD*-like gene, as no *rpmD* has been annotated on the JCVI-syn3.0 genome, although it is present in all minimal sets we are working with, an indication that it must be essential. Moreover, a putative duplicated *pheT* gene was found. Figure 3 shows the comparison among the three translational datasets. All three datasets, share 95 genes, thus confirming that not all the genes needed are strictly essential (Acevedo-Rocha et al., 2013). Finally, of the 147-genes identified as components of the JCVI-syn3.0 translation machinery, 106 genes (72.1%) are shared with our new proposal for minimal a translation machinery and correspond mainly to ribosomal proteins and aminoacylation enzymes (Supplementary Material 4).

Lastly, based on information from the BioCyc and UniProt databases, we performed a functional analysis of the genes found in JCVI-syn3.0 that had not been included in our first proposal, to improve the efficiency of the translational machinery model. Furthermore, through this functional analysis we checked if any function from our first proposal was unnecessary or redundant. For example, in the cases in which there were specific genes of Gram-negative or Gram-positive bacteria (i.e., non-orthologous gene displacement), since this study is based mainly on data from Gram-negative bacteria, we chose to include the alternative corresponding to this group. Thus, regarding aminoacyl-tRNA synthetases (EC 6.1.1.-), our proposal has included *glnS* and *gltX* instead of *gluS*, *gatA*, *gatB*, and *gatC*. As for the tRNA modification genes, we think that only *thiI* and *tsaE* need to be added. Both are related to the modifications at position 8 and 37 of tRNA, respectively. ThiI (EC 2.8.1.4) is an enzyme that produces 4-thiouridine [s(4)U8] (Kambampati and Lauhon, 2000), and it is encoded by an essential gene in *E. coli* K-12 MG1655 (Rajakovich et al., 2012). Moreover, IscS-IscU (EC 2.8.1.7 and EC 3.6.4.10, respectively), two partners of ThiI involved in biological iron–sulfur cluster assembly, needed for sulfur transfer, are already included in our proposal, are essential in both *E. coli* and *B. subtilis*, and are present in JCVI-syn3.0 genome. Finally, we consider that *iscA* should be included as well, although it is not in any of the results shown so far (i.e., JCVI-syn3.0, DEG or our first proposal). This decision is because IscA is necessary for the proper operation of the IscS-IscU system. Although any other alternative enzyme of the HesB family could replace it (López-Madrigal et al., 2013), at least one of them must be part of the minimal translation machinery. On the other hand, TsaE is involved in the formation of a threonylcarbamoyl group (t_6_A37), a universally conserved modification (Thiaville et al., 2015). It acts with TsaB, TsaC (EC 2.7.7.87) and TsaD (EC 2.3.1.234), encoded by essential genes included in DEG, and present both in our first proposal and in the JCVI-syn3.0 genome. Regarding to the subcategory translation, we added *tufA* and *tufB* genes, both responsible for the formation of the EF-Tu protein whose essential function is delivering aminoacylated tRNA into the A-site of the ribosome during protein biosynthesis (Kacar et al., 2017). In addition of being necessary for proper translation, they are considered essential in the DEG databases, and are present in the JCVI-syn3.0 genome. As for the subcategory Ribosome assembly, we think that *rimM* must be added because, together with *rbfA*, is needed for efficient processing of 16S rRNA in *E. coli* (Bylund et al., 1998). The *cca* gene (tRNA aminoacylation) which encodes the enzyme Ccase (EC 2.7.7.72) that adds and repairs the 3’-terminal CCA sequence in tRNAs is not included in JCVI-syn3.0. In our results this gene is present to compensate the tendency to have tRNA without the CCA end in OS genomes (data not shown). Finally, nine ribosomal proteins that are present in the JCVI-syn3.0 genome are not include in our first proposal: *rplJ* (L10), *rpmC* (L29), *rpmD* (L30), *rpmE* (L31), *rpmF* (L32), *rpmJ* (L36), *rpsP* (S16), *rpsR* (S18), and *rpsU* (S21). It is known that the composition of the large subunit of the ribosome is less conserved than the one of the small subunit. Moreover, genes of S21, L30 and L31 ribosomal proteins have been consistently reported to be missing (Nikolaeva et al., 2021). Based on these facts, we have only added *rpsP* and *rpsR* to our minimal translation machinery.

**FIGURE 4:**
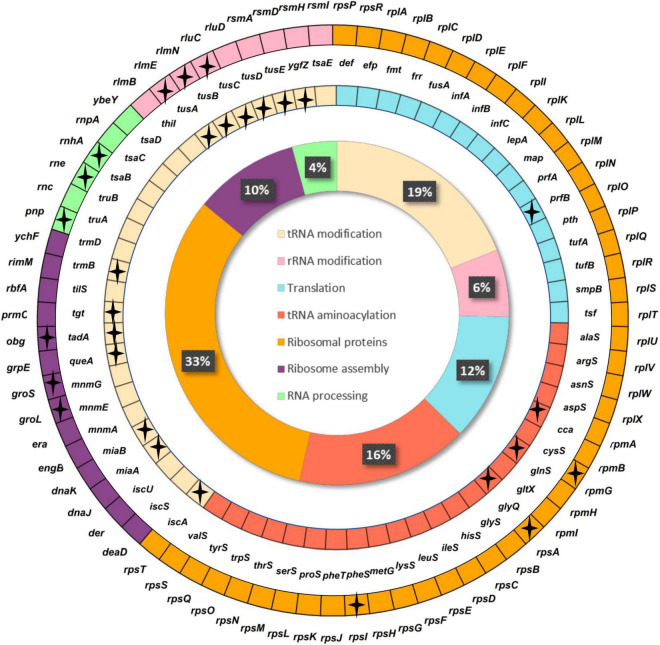
Minimal translational machinery proposed in this work. Genes are classified by translational subfunction. Inner circle shows the percentage of genes in each subcategory based on hierarchical cluster analysis (HCA) analysis. Stars indicate genes not found in JCVI-syn3.0.

The final minimal translation machinery proposed in our study (Figure 4) is composed of 142 genes, 113 of which are shared with JCVI-syn3.0 (76.8%), while 112 and 87 (78.8 and 61.3%) are essential in *E. coli* K-12 MG1655 and *B. subtilis* 168, respectively.

## Discussion

Based on a computational comparison of genomic information about model and highly reduced bacterial genomes available in public databases plus modern machine learning techniques, we propose a minimal translational gene-set that consists of 142 genes. This work goes beyond the previous proposal of a minimal translation machinery established in Mollicutes (Grosjean et al., 2014), as it includes information about both Gram-positive and Gram-negative organisms with naturally and artificially-reduced genomes, plus a thorough manual curation of all the conjoined information to search for possible mislead or missed genes to define the minimal gene-set implied in a universal translation process. Nevertheless, there is broad agreement with the minimal translational machinery defined by Grosjean et al. (2014).

Ribosomal proteins are part of the ribosome together with rRNAs. In our model, we include 46 genes out of 54 related to this category. Of these, 43 are included in the JCVI-syn3.0 genome and 49 are largely conserved in Mollicutes (Grosjean et al., 2014). It has been experimentally described that at least half of the ribosomal proteins in *E. coli* and *B. subtilis* are not essential for cell survival when individually deleted (Shoji et al., 2011; Akanuma et al., 2012). The absence of some ribosomal proteins in our set can be explained by two non-exclusive reasons. It cannot be discarded that, due to their small size, some apparently absent ribosomal genes are present but have not been detected (i.e., annotated) in the genomes under study. Otherwise, they could have been in fact lost in tiny bacterial genomes, which tend to lose mainly proteins of the large subunit located on the surface of the ribosome (Galperin et al., 2021; Nikolaeva et al., 2021). Regarding this point, seven genes that have not been included in our proposal code for large ribosomal subunit components. Altogether, the ribosomal proteins we have included in our proposal are consistent with the theoretical or experimental ribosomal-proteins sets described in the literature.

Translation factors perform diverse functions throughout the translation process, optimizing it. Most of them were conserved in the 39 Mollicutes genomes studied. Of the 51 genes from the universal set under study that belong to the category Translation, 17 are included in our proposal, and match genes present in the JCVI-syn3.0 genome except for *prfB*, a gene that is absent in most Mollicutes. It codes for release factor 2 (RF-2), the one that recognizes the UAG codon, which is recoded from stop to tryptophan in most of these clade members (Grosjean et al., 2014).

Ribonucleases (RNases) that process different RNA precursors are also needed in translation. Only three RNases were found in all Mollicutes genomes analyzed by Grosjean et al. (2014), which indicates that most of them do not need to be included in the minimal translational gene-set. In our analysis, we have found six genes included in the category RNA processing, only three of which are coincident with JCVI- syn3.0, but not with those found in Mollicutes, thus confirming that ribonucleases are not conserved uniformly in the naturally reduced genomes.

Regarding aminoacyl-tRNA synthetases (EC 6.1.1.-), the enzymes that add the corresponding amino acid to the appropriate tRNA, it has been described that only one of them is conserved for each amino acid in small genomes, while tiny genomes do not retain a whole set, probably because some of these enzymes can exert multifunction (i.e., an aminoacyl-tRNA synthetase can load several different amino acids) (Moran and Bennett, 2014) or because they can borrow those from a cosymbiont or the host (Reyes-Prieto et al., 2014). Grosjean and colleagues found 20 aminoacyl-tRNA synthetases were preserved in Mollicutes, some of which are multimeric, and are not coincident to those found in Gram-negative bacteria (Gil et al., 2004). Thus, based on the first *in silico* proposal, taking into account that the *E. coli* model was our default selection in case of divergence, and to warrant an efficient functioning of the system, we have maintained all 21 genes included in this category in previous studies based on endosymbiont genomes (Gil et al., 2004), which include 20 of the 33 genes of this category found in the Mollicutes dataset, plus *cca* and *glyQ.* The latter, which was not included in previous minimal genome proposals (Gil, 2014), encodes the alpha-subunit of glycyl-tRNA synthetase, and was added because both subunits appear to be necessary for its proper functioning in *E. coli* (Ju et al., 2021).

A large part of the genes involved in the post-transcriptional modifications of tRNAs and rRNAs have been lost in small genomes (Hansen and Moran, 2012). In fact, this is also the category in which greater diversity is observed in Mollicutes (de Crécy-Lagard et al., 2012; Grosjean et al., 2014). Therefore, these genes appear to be dispensable for a minimal translational machinery, even considering the importance of the chemical modification of specific bases in the structure and function of these RNAs. These modifications have been detected in all domains of life (Sergiev et al., 2011) and can be simple (e.g., methylations, thiolations, pseudouridinations) or complex, including the addition of an amino acid (glycine, taurine, threonine, etc.) in tRNAs. Specifically, the modifications of the anticodon domain let the three-dimensional structure of each tRNA to be set for its proper positioning in the ribosome (Agris, 2008), thus facilitating the correct mRNA decoding. The modifications in positions 34 and 37 are fundamental for this purpose, and it is precisely where the greatest variety of modifications are found (Armengod et al., 2014). The minimal translational gene-set defined in Mollicutes revealed that several tRNA modifying enzymes have not been lost due to genome reduction (Grosjean et al., 2014). Yet, the number of genes involved in tRNA modifications decreases drastically along with genome size in obligate endosymbionts, including some of the genes coding for factors involved in these modifications, such as MnmA (EC 2.8.1.13) and the IscS (EC 2.8.1.7), TrmD (EC 2.1.1.228) and TsaC (EC 2.8.1.7) complexes. Little is known to date about tRNAs modification in endosymbionts because, as they cannot be cultured in the laboratory, it is not possible to analyse the state of modification of tRNAs with conventional techniques. The ongoing renewal and improvement of the latest generation of sequencing techniques are making them a valuable tool to deepen in their study (Zhang et al., 2022). Nevertheless, it has been proposed that the tRNAs from endosymbiont such as *B. aphidicola* must have specific changes in the critical bases that would stabilize the structure of the molecule to exercise its translational function despite having A + T rich sequences (Hansen and Moran, 2012). It is currently unknown if these endosymbiont organisms use any of the host’s modifying enzymes or if they can use their own modifying enzymes that have not yet been identified, which would improve the structural state of tRNA to optimize its translational function. After adding all these genes for optimal performance, our proposal includes 27 genes for post-transcriptional modifications, 14 of which match the JCVI-syn.3.0 genome, and 10 match the Mollicutes translation machinery proposal.

As for the modifications of rRNAs, they help the translation process by different mechanisms. Many of them are involved in stabilizing the three-dimensional structure of the ribosome, and they are mostly concentrated around the ribosomal catalytic sites. In addition, they promote the interaction with ligands during the translation process and act as checkpoint marks for control of the process (Sergiev et al., 2011). While there are about 36 rRNA modifications in *E. coli*, only 14 have been described in species of the genus *Mycoplasma* (de Crécy-Lagard, 2007). We retrieved nine genes in our proposal, six out of which are present in JCVI-syn3.0.

Since the JCVI-syn3.0 genome has been experimentally minimized and its viability as an organism has been proven, the 113 orthologous genes shared between this genome and our proposal must be essential. The differences between them can be explained by several non-exclusive causes, including non-orthologous gene displacement, adaptation to different environments and protein multifunctionality.

It is widely accepted that the environment strongly influences what would be essential genes for a minimal cell (Koonin, 2003; Gil and Peretó, 2015). Because our main data source are insect symbionts, the environment is very different from that of a free-living cell or one grown in the laboratory under controlled conditions. This could be reflected in the need of different RNA modifying and RNA processing enzymes, where greater differences were found in our comparisons. Remarkably, 16 of the 29 genes included in our first proposal that are not present in JCVI-syn3.0, code for rRNA/tRNA modifying enzymes. These results suggest that the acquisition (or retention) of RNA-modifying enzymes could play an enriching role for bacteria to survive in different environments. From a biotechnological point of view, these alternative enzymes could be tested to build biological systems adapted to specific environmental conditions. In addition, it remains to be determined whether a non-redundant genetic code would make some tRNAs modifications unnecessary.

We have worked with the concept one gene-one function, which is known to be inaccurate. It cannot be ruled out that proteins with low substrate specificity could replace the function of others (e.g., a specific methylase could be able to methylate non-specifically other substrates), while it is known that many proteins can be involved in more than one, sometimes unrelated, functions (moonlighting proteins; Shirafkan et al., 2021). In addition to experimental validation of genome reductions such as the one achieved with *M. mycoides* JCVI-syn3.0, a better delineation of what should be the minimal number of genes necessary to obtain a simplified but still efficient bacterial translational apparatus can be achieved by using machine-learning methods to detect replacements and moonlighting scenarios.

## Data Availability Statement

The original contributions presented in the study are included in the article/Supplementary Material, further inquiries can be directed to the corresponding author/s.

## Author Contributions

RG and MG: conceptualization. MG and MR-P: data curation, orthology analysis, and validation. RG: supervision, funding acquisition, and project administration. MG: writing original draft. All authors contributed to the formal analysis and visualization and reviewed, edited, and approved the final manuscript.

## Conflict of Interest

The authors declare that the research was conducted in the absence of any commercial or financial relationships that could be construed as a potential conflict of interest.

## Publisher’s Note

All claims expressed in this article are solely those of the authors and do not necessarily represent those of their affiliated organizations, or those of the publisher, the editors and the reviewers. Any product that may be evaluated in this article, or claim that may be made by its manufacturer, is not guaranteed or endorsed by the publisher.

## Footnotes

1
https://www.ncbi.nlm.nih.gov/genome/

## Supplementary Material

Supplementary Figure 1 Dendrograms obtained as a result of HCA for cosym dataset. The analyses have been performed for each translational subprocess (legend A) considering clades (legend B).

Supplementary Material 1 Genomes and cosymbionts of the study (XLSX file). “Genomes” sheet: List of organisms with the abbreviation code used in this study. “Consortia” sheet: list of cosymbionts (symbiotic consortia) used as unique genome entity.

Supplementary Material 2 Translational genes set (XLSX file). List of genes with translational functions used as universe in this study.

Supplementary Material 3 Matrix of presence/absence of genes in the 92 genomes of the study (XLSX file). Zero values indicate absence of the gene.

Supplementary Material 4 Comparison among cosym, *bcc*, *ssz*, JCVI-syn3.0, and DEG datasets (*Escherichia coli* and *Bacillus subtilis*) used in the study (XLSX file). The abbreviation code is the same as in Supplementary Material 1.
