# Supplementary material for: The Minimal Translation Machinery: What We Can Learn From Naturally and Experimentally Reduced Genomes
Source: Front Microbiol. 2022 Apr 11;13:858983. doi: 10.3389/fmicb.2022.858983 (PMC9035817; doi:10.3389/fmicb.2022.858983)
Supplement: Supplementary Figure 1 — Dendrograms obtained as a result of HCA for cosym dataset. The analyses have been performed for each translational subprocess (legend A) considering clades (legend B). [file Image_1.pdf]

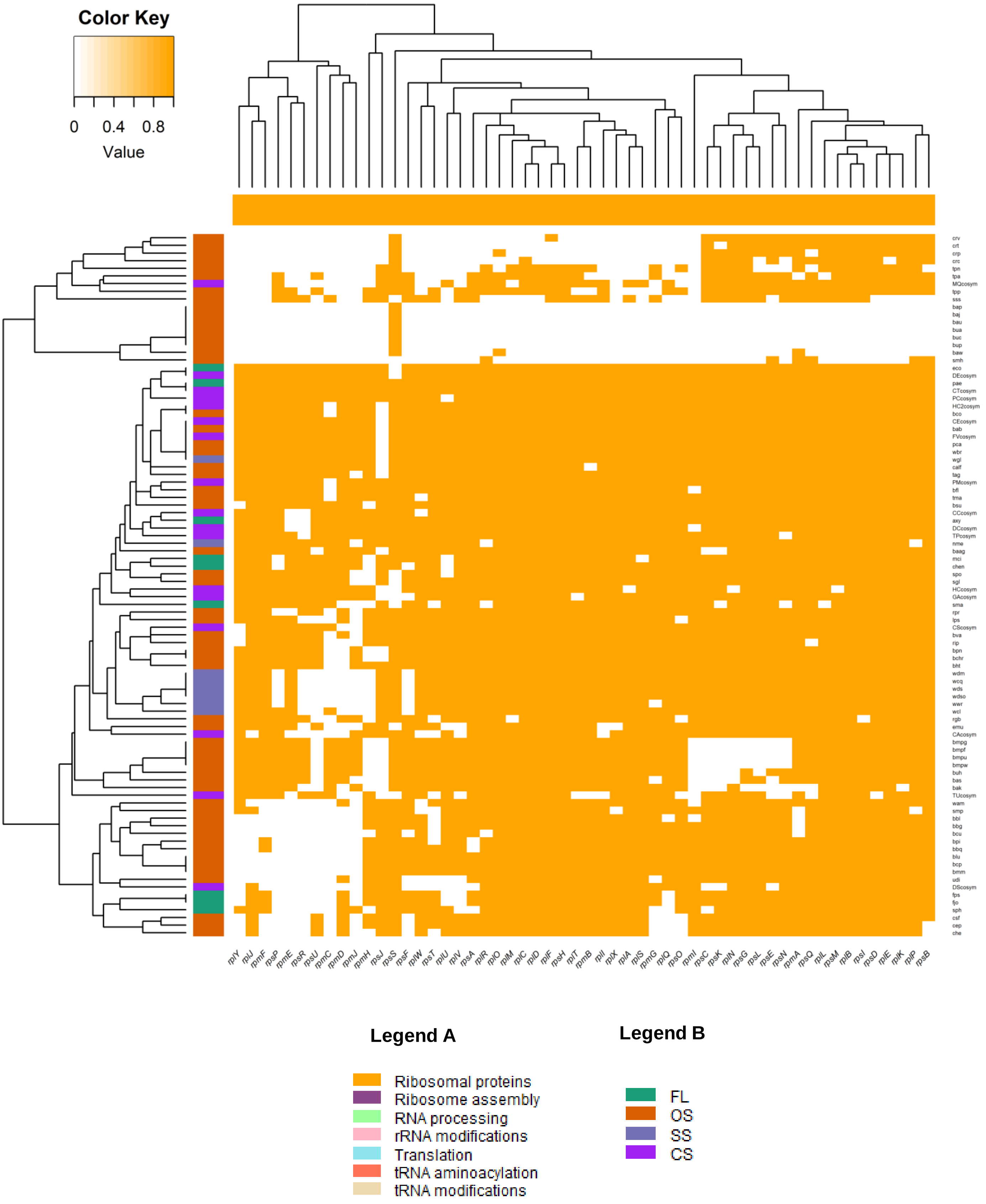

Supplementary Figure 1. Dendrograms obtained as a result of HCA for cosym dataset. The analyses have been performed for each translational subprocess (legend A) considering clades (legend B).

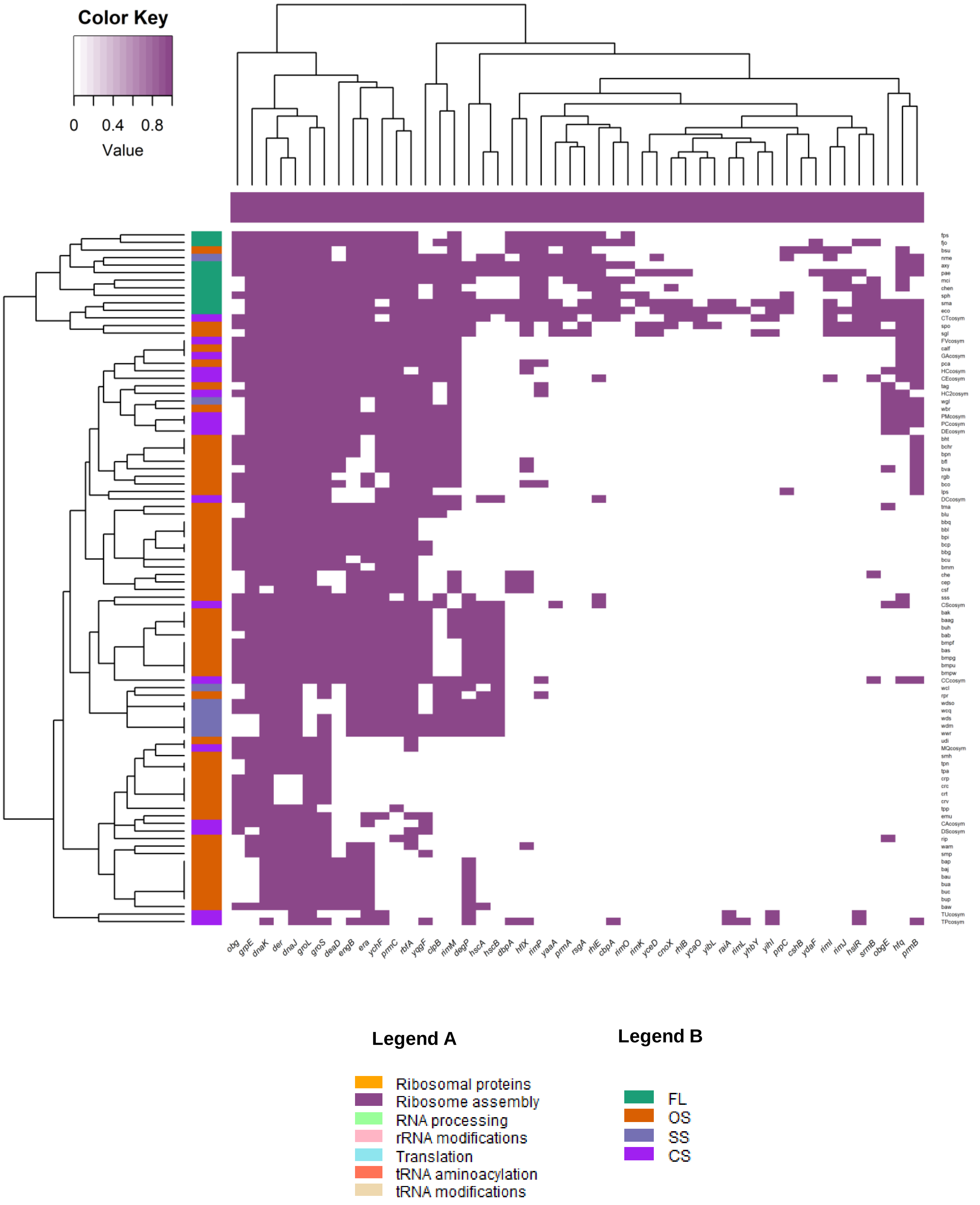

(Continued) Supplementary Figure 1. Dendrograms obtained as a result of HCA for cosym dataset. The analyses have been performed for each translational subprocess (legend A) considering clades (legend B).

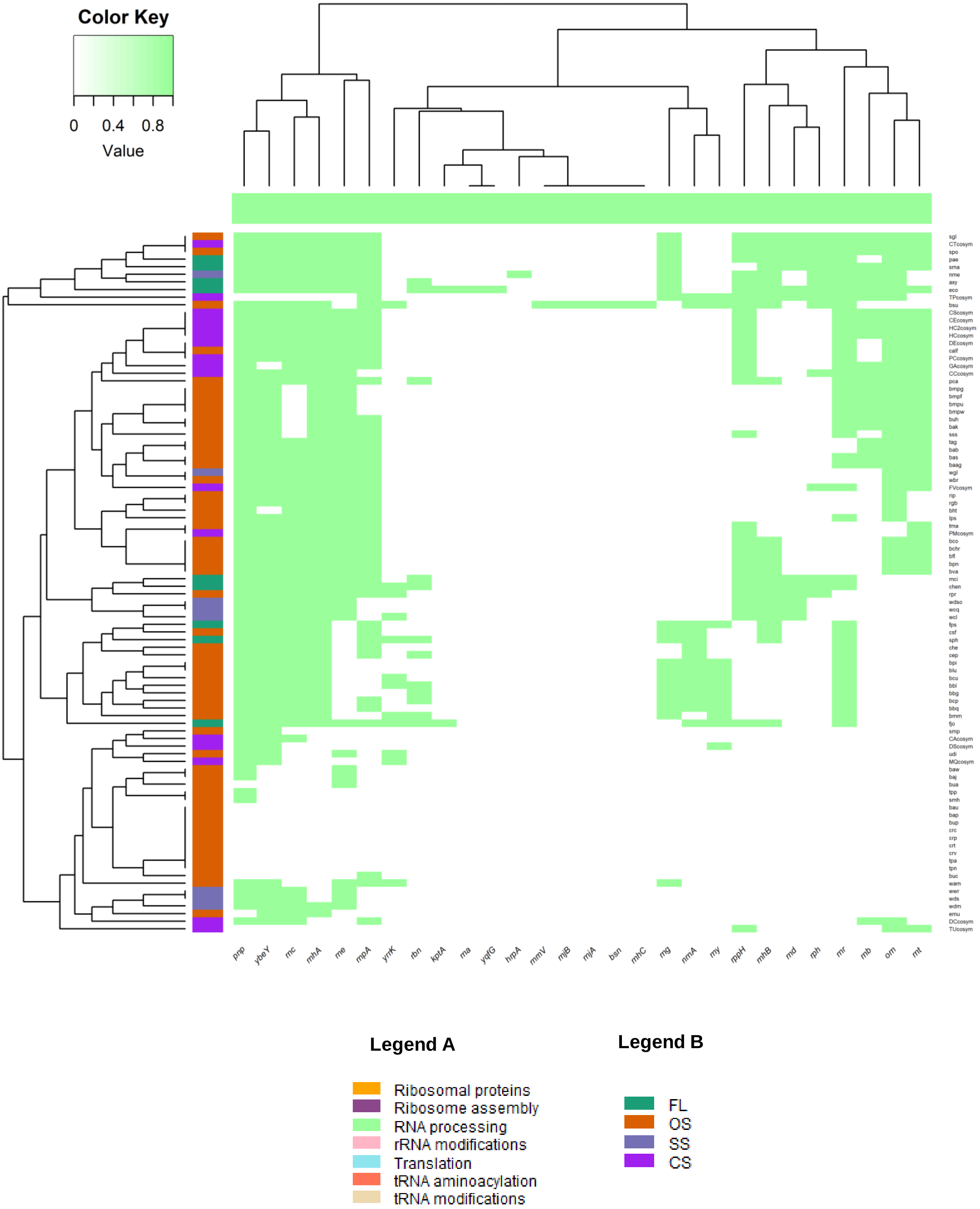

(Continued) Supplementary Figure 1. Dendrograms obtained as a result of HCA for cosym dataset. The analyses have been performed for each translational subprocess (legend A) considering clades (legend B).

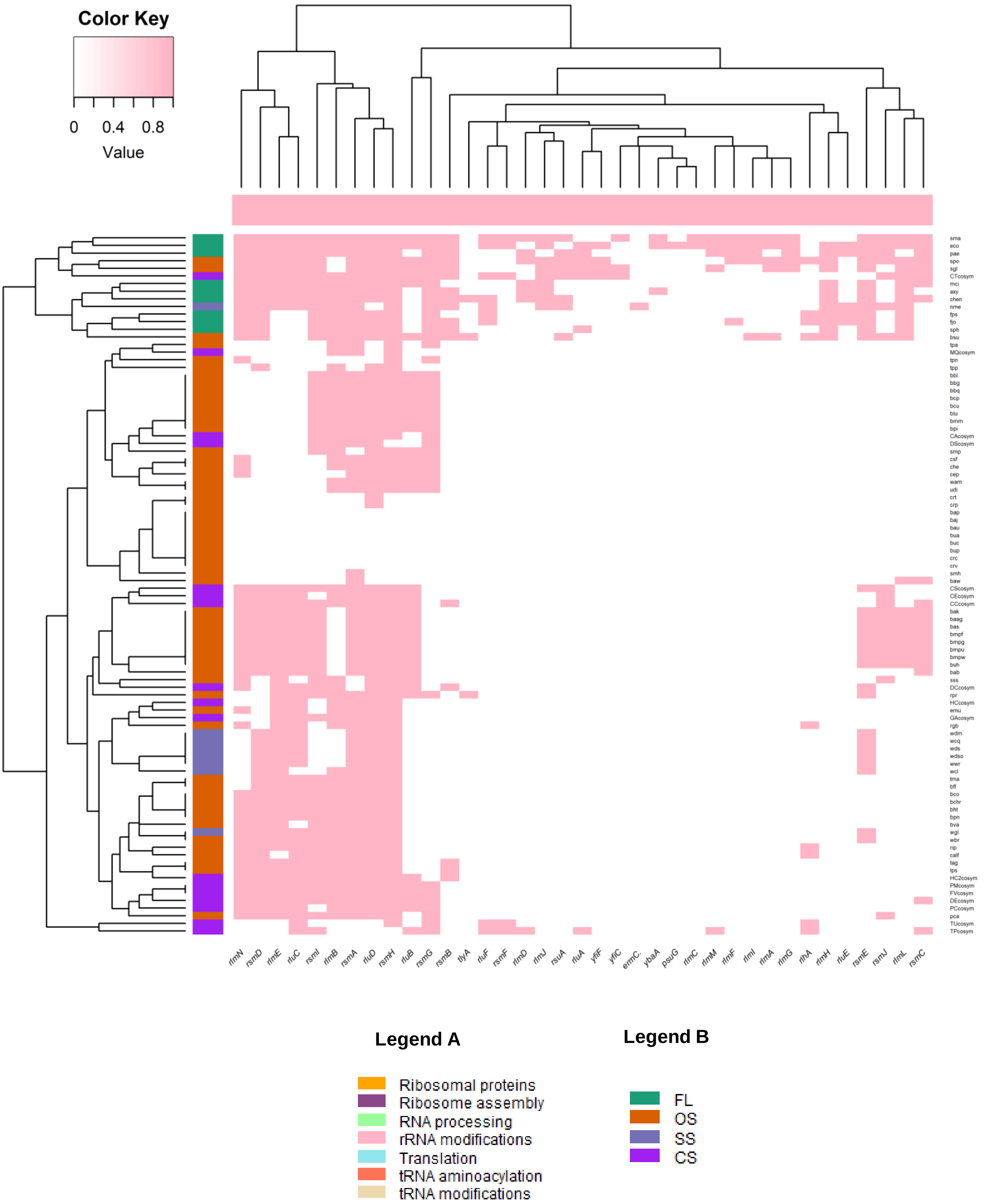

(Continued) Supplementary Figure 1. Dendrograms obtained as a result of HCA for cosym dataset. The analyses have been performed for each translational subprocess (legend A) considering clades (legend B).

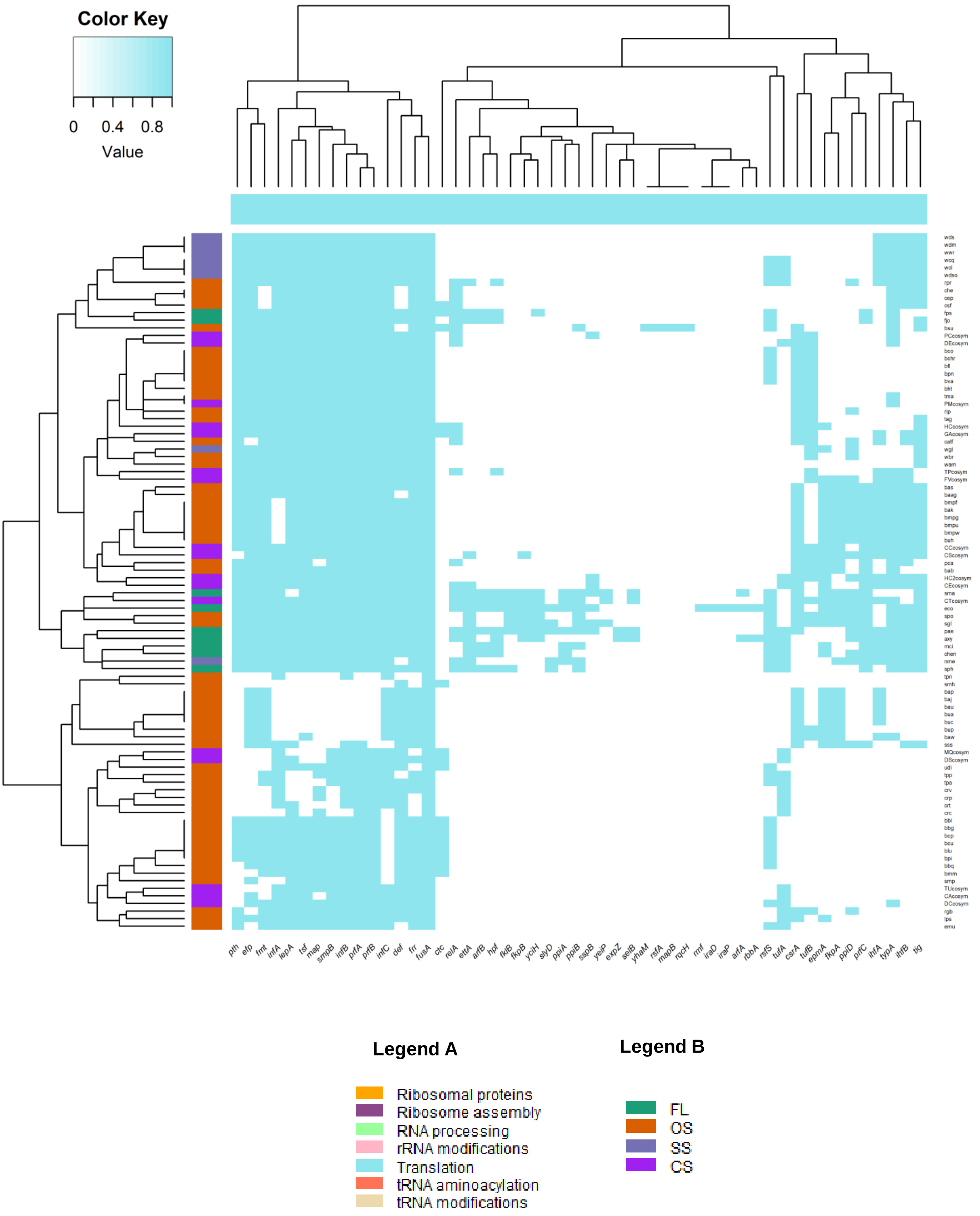

(Continued) Supplementary Figure 1. Dendrograms obtained as a result of HCA for cosym dataset. The analyses have been performed for each translational subprocess (legend A) considering clades (legend B).

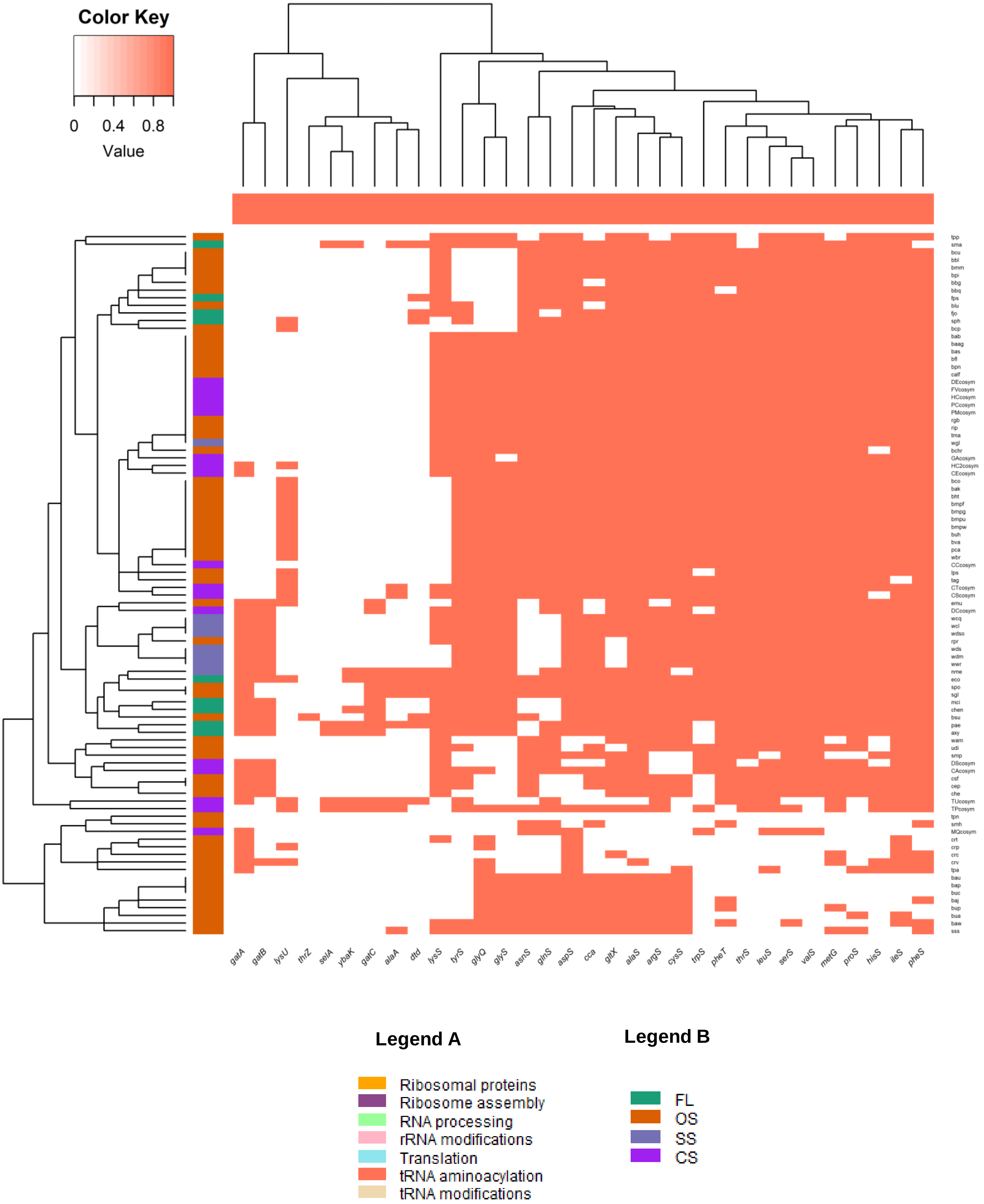

(Continued) Supplementary Figure 1. Dendrograms obtained as a result of HCA for cosym dataset. The analyses have been performed for each translational subprocess (legend A) considering clades (legend B).

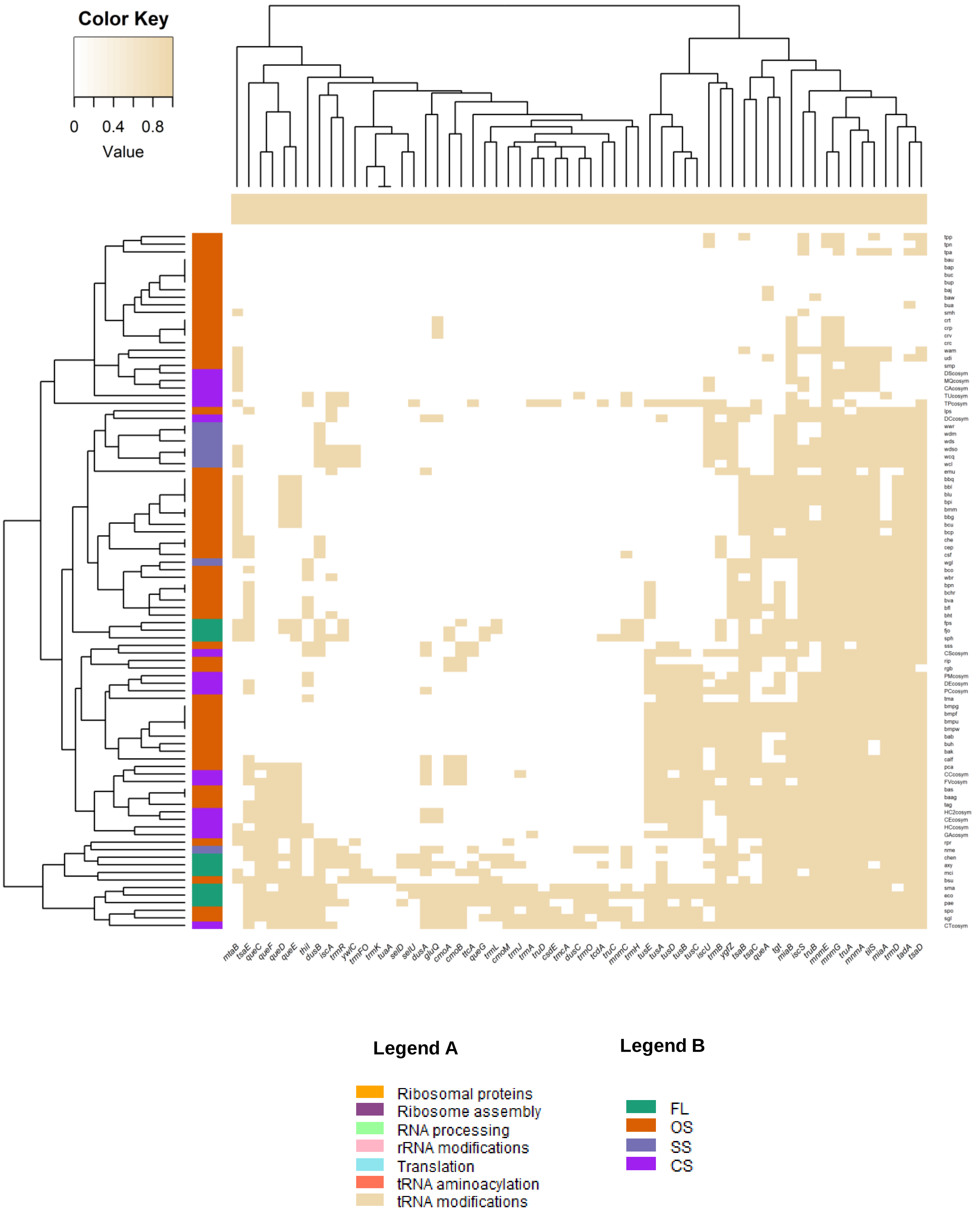

(Continued) Supplementary Figure 1. Dendrograms obtained as a result of HCA for cosym dataset. The analyses have been performed for each translational subprocess (legend A) considering clades (legend B).
